# Supplementary material for: Machine learning discovers numerous new computational principles supporting elementary motion detection
Source: Nat Commun. 2026 Mar 3;17:3424. doi: 10.1038/s41467-026-70288-4 (PMC13076616; doi:10.1038/s41467-026-70288-4)
Supplement: Supplementary file 1 — Supplementary Information [file 41467_2026_70288_MOESM1_ESM.pdf]

## Supplementary figures

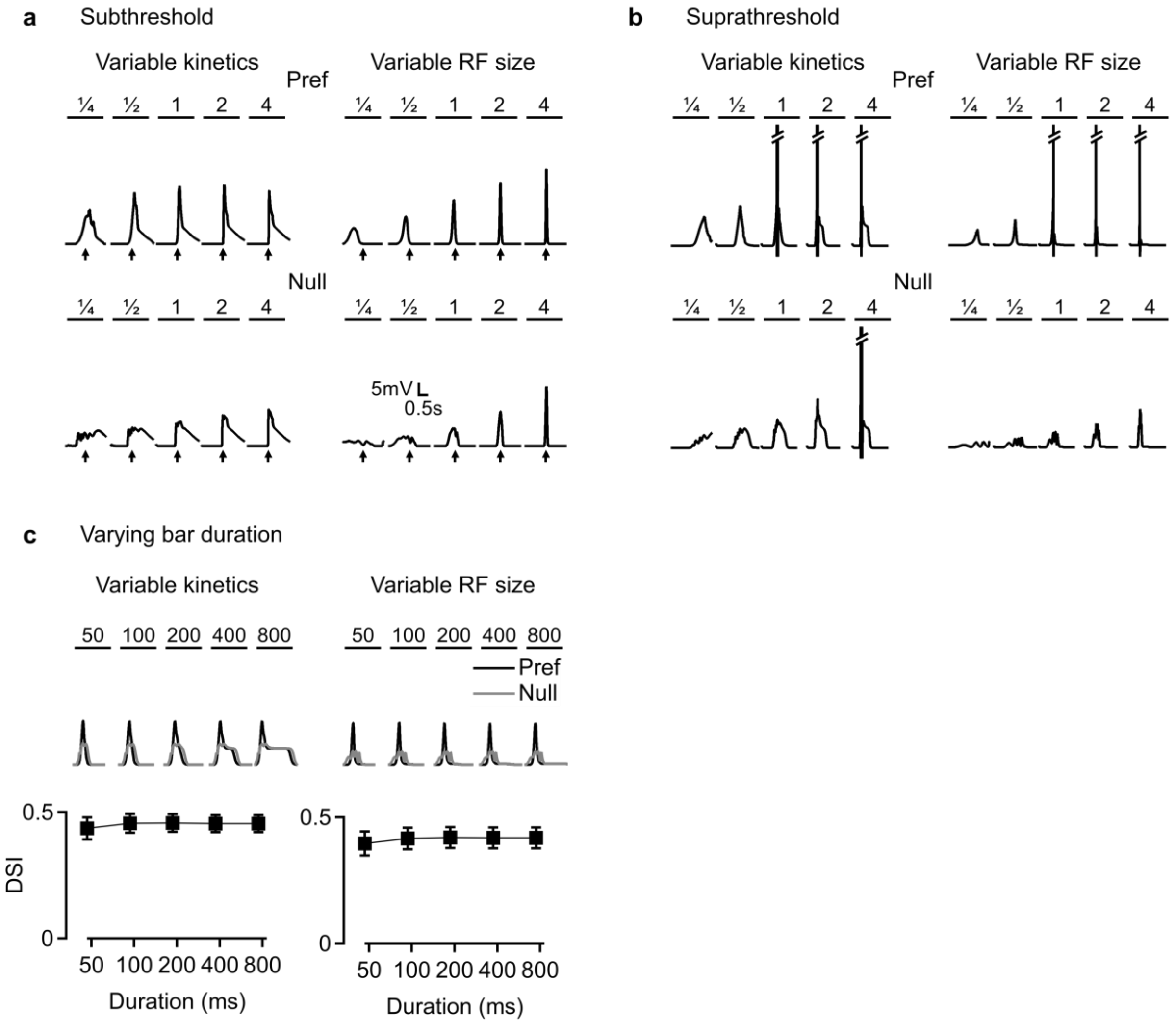


**Supplementary Figure 1: Robust direction selectivity in models with varying center components of presynaptic receptive fields.**

**a**, Somatic voltage responses (top, preferred direction; bottom, null direction) across multiple velocities for the models in (**Fig. 1b, c**). The spatial RF model exhibits earlier peak times. Arrows mark the time when the stimulus reaches the position of DSGC soma. **b**, Same conditions as in (**a**), but models were first trained to maximize subthreshold responses. Somatic sodium and potassium channels were then introduced into the DSGC model, enabling evolution of suprathreshold DS. **c**, Top, as in (**a**), but for models trained to produce DS with bars of different durations (speed = 1 mm/s). Bottom: summary of 100 trials with unique randomized seeds showing comparable DSI values across bar durations (error bars, SD).

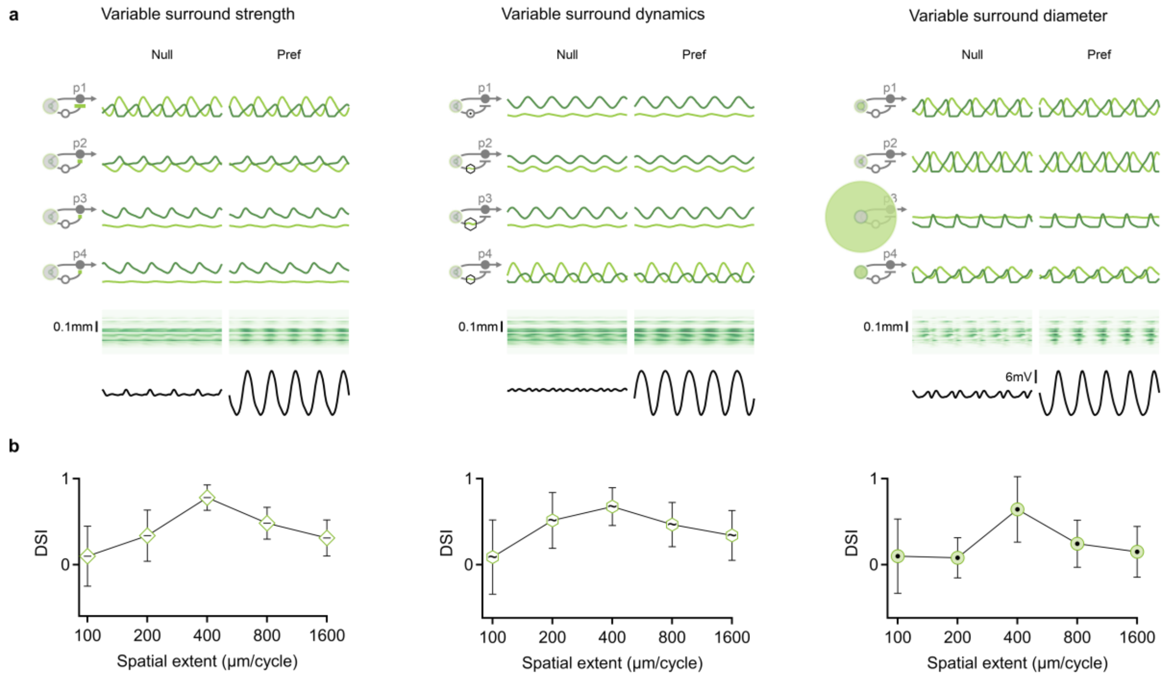


**Supplementary Figure 2: Models with variable receptive field surrounds responding to drifting gratings.**

DS performance of models trained to optimize response to drifting gratings stimuli (five different spatial frequencies), constrained to vary only in the strength (left), kinetics (center), or size (right) of the surround RF component. **a**, Top, RF center (dark green) and surround (light green) responses in the four presynaptic populations (spatial extent of the gratings = 400 µm/cycle). Center, activation profiles of the presynaptic inputs, as in **Fig. 2d**, illustrating temporal synchronization during preferred direction motion (right). Bottom, postsynaptic responses. **b**, Mean (±SD) directional performance for different spatial extents of the drifting gratings (n = 30 independently randomized simulation runs).

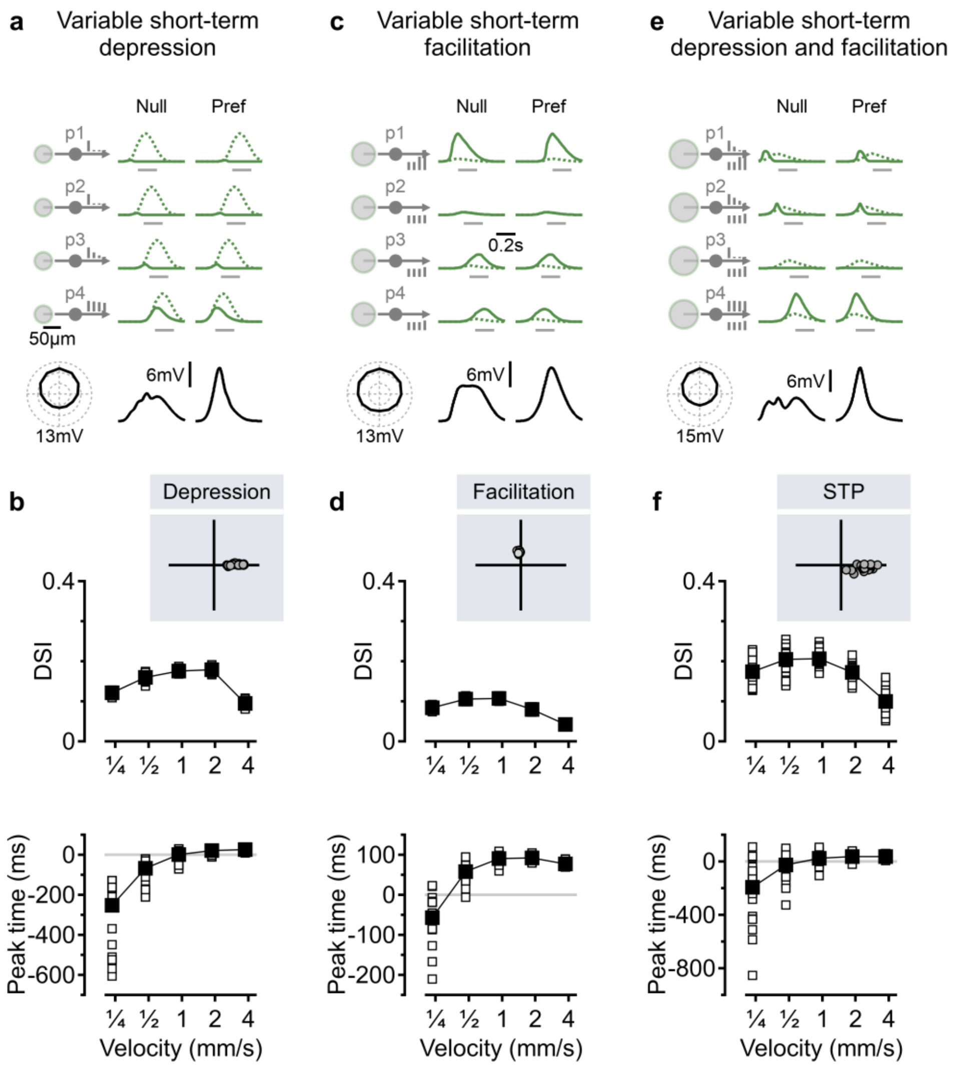


**Supplementary Figure 3: Models with short-term plasticity of the excitatory drive.**

**a**, Example solution for DS circuit composed of presynaptic excitatory cells with identical RFs but different short-term synaptic depression levels. Dotted, RF activation prior to synaptic depression. The level of synaptic depression is depicted schematically for each input population. **b**, Velocity tuning (top) and peak response times (bottom). Inset, computational primitives, or algorithmic solutions seen in each of the model families. See (**Fig. 4**) for color-coding and axis details. **c-d**, As in (**a-b**), but for varying short-term facilitation. **e-f**, As in (**a-b**), but for models allowed to vary in both short-term facilitation and depression (n = 30 independently randomized simulation runs).

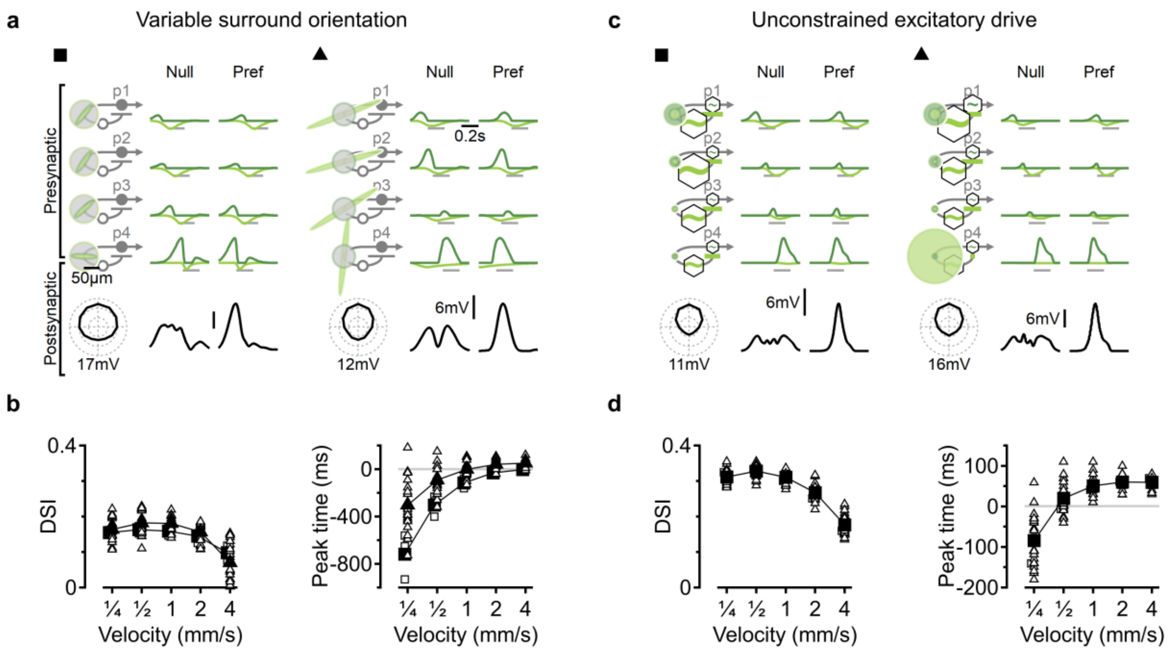


**Supplementary Figure 4: Direction selectivity in models with varying presynaptic receptive field surround orientation or unconstrained excitatory drive.**

**a**, Two example solutions for the direction selectivity circuit composed of presynaptic cells with varying surround orientations. Spatiotemporal characteristics of the center, surround temporal dynamics, and the spatial shape of the surround are identical between the inputs. **b**, Velocity tuning curves (left) and peak response times (right) of all optimal solutions (open symbols) and cluster means (black symbols and connecting lines); cluster assignment was calculated using hierarchical clustering. (**c**-**d**) As in (**a**-**b**) for selectivity circuit models where all spatiotemporal RF components were allowed to vary between the presynaptic populations ( n= 30 independently randomized simulation runs).

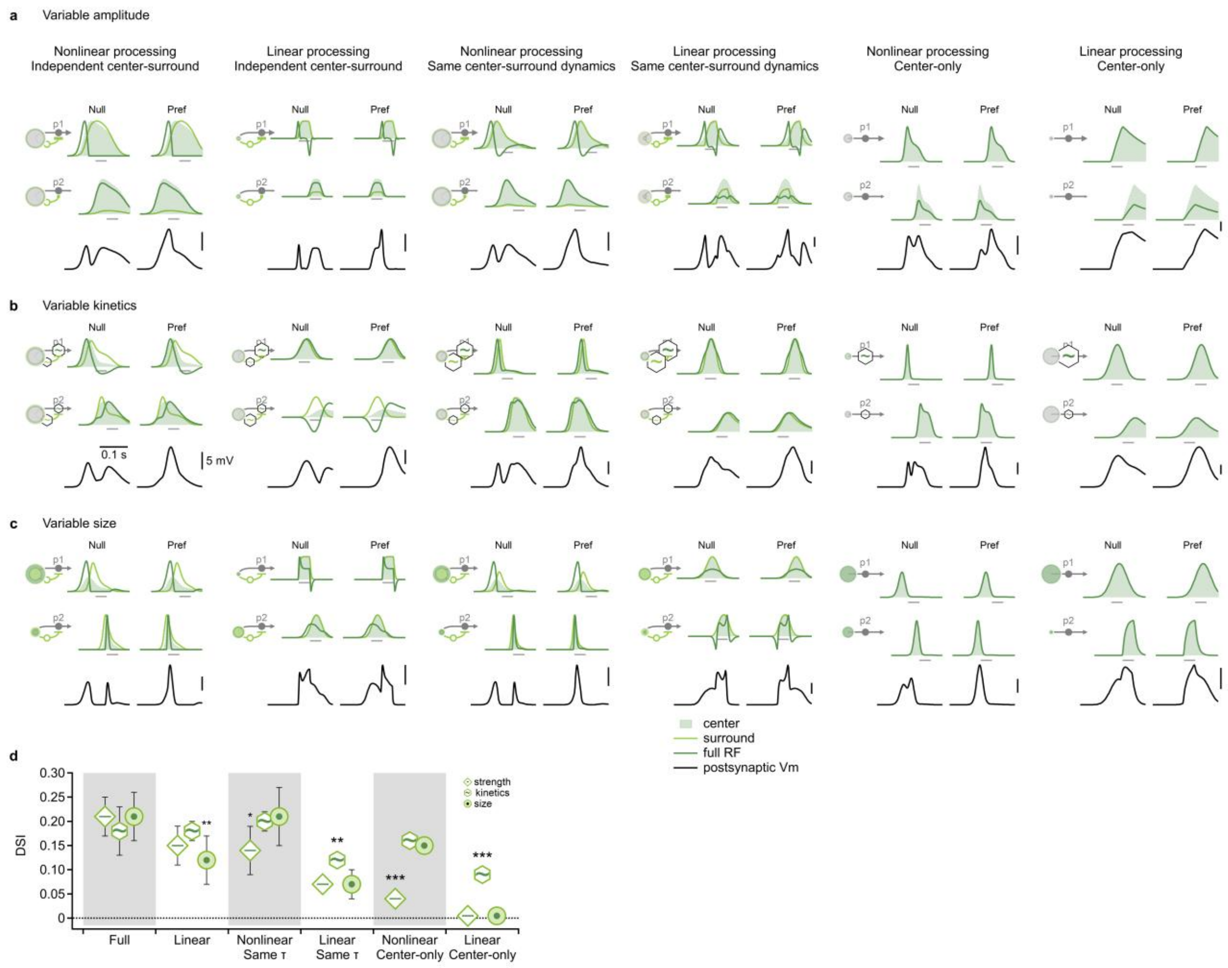


**Supplementary Figure 5. Receptive field components mediating direction selectivity for moving bar stimulation.**

**a-c**, Representative responses from evolved models containing two presynaptic inputs positioned 200 µm apart and converging onto a single passive postsynaptic compartment. Models were constrained such that the two inputs could differ only in amplitude (a), kinetics (b), or spatial extent (c) of their RFs. Each column shows a distinct RF formulation: (1) Full nonlinear center-surround RF, incorporating synaptic adaptation via RRP depletion and rectification of the combined center-surround signal; (2) Linear center-surround RF, where spatial activation of each RF component is convolved with its temporal filter; (3) Nonlinear release with matched center and surround kinetics; (4) Linear release with matched center and surround kinetics; (5–6) Center-only RFs, with or without synaptic adaptation. Schematics depict the spatial and temporal RF properties of evolved solutions. Response waveforms: center (filled green), surround (light green), and combined center-surround (dark green). Bottom: postsynaptic response from both inputs (black). **d**, Mean ± SD direction selectivity index for each RF configuration under the three model constraints (N = 100 independently randomized simulation runs per model). *$p < 0.05$, **$p < 0.01$, ***$p < 0.001$; ANOVA with Bonferroni correction. Amplitude- and size-based models did not produce DS for the linear center-only configuration.

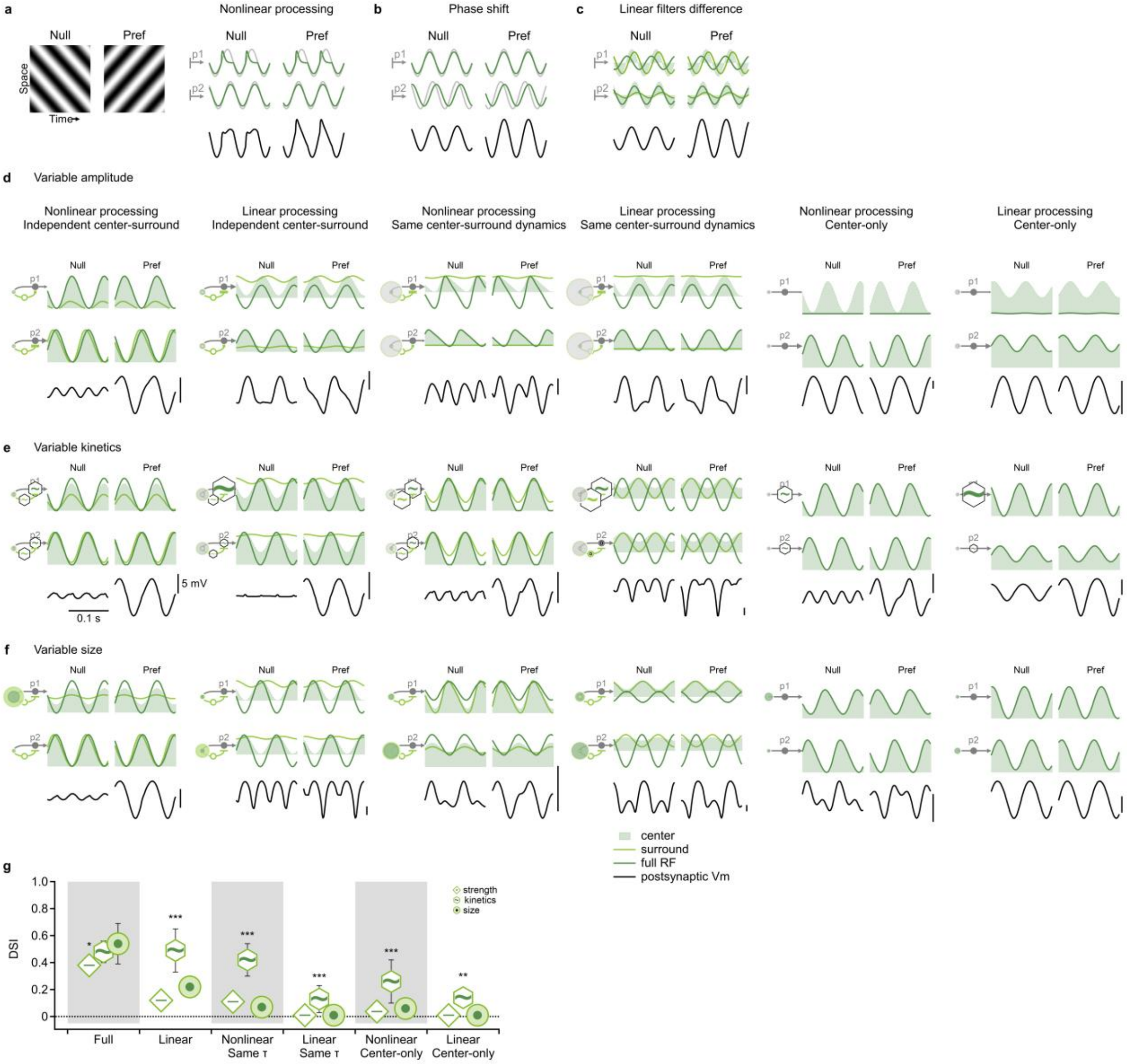


**Supplementary Figure 6. Receptive field components mediating direction selectivity for sinusoidal drifting gratings.**

**a**, Left: space-time plot of a drifting sinusoidal grating stimulating two inputs, as in **Supplementary Fig. 5**. The model was trained to produce a larger steady-state response (peak-to-trough amplitude) for upward motion. Three algorithmic classes of DS solutions can emerge in this circuit: Nonlinear signal processing, which distorts the RF response so that it deviates from a pure sinusoid (**a**, right); (**b**) Linear phase shifts in the RF response generated directly by the RF kinetics; (**c**) Phase shifts arising from center-surround interactions, where integration of two sinewaves with different temporal filters alters the composite phase. In all examples, center responses are shown in filled green, surround responses in light green, and the combined center-surround signal in dark green. Bottom: linear summation of the two inputs (black). **d-f**, As in **Supplementary Fig. 5a-c**, but stimulated with drifting sinusoidal gratings rather than moving bars. **g**, Mean ± SD direction selectivity for each RF configuration shown in (**d–f**) (N = 100 independently randomized simulation runs per condition). *$p < 0.05$, **$p < 0.01$,

***p < 0.001 (ANOVA with Bonferroni correction). Amplitude- and size-based models did not produce DS for linear RFs with matched center-surround kinetics or in center-only configurations.

As shown, kinetics-based solutions reliably support DS because their temporal asymmetries directly shift the phase of the response. In contrast to moving-bar stimuli, RF size alone modifies only the response amplitude, not shape. Thus, models constrained to vary only RF amplitude or spatial extent required either nonlinear integration or center-surround components with distinct kinetics to generate DS for drifting gratings.

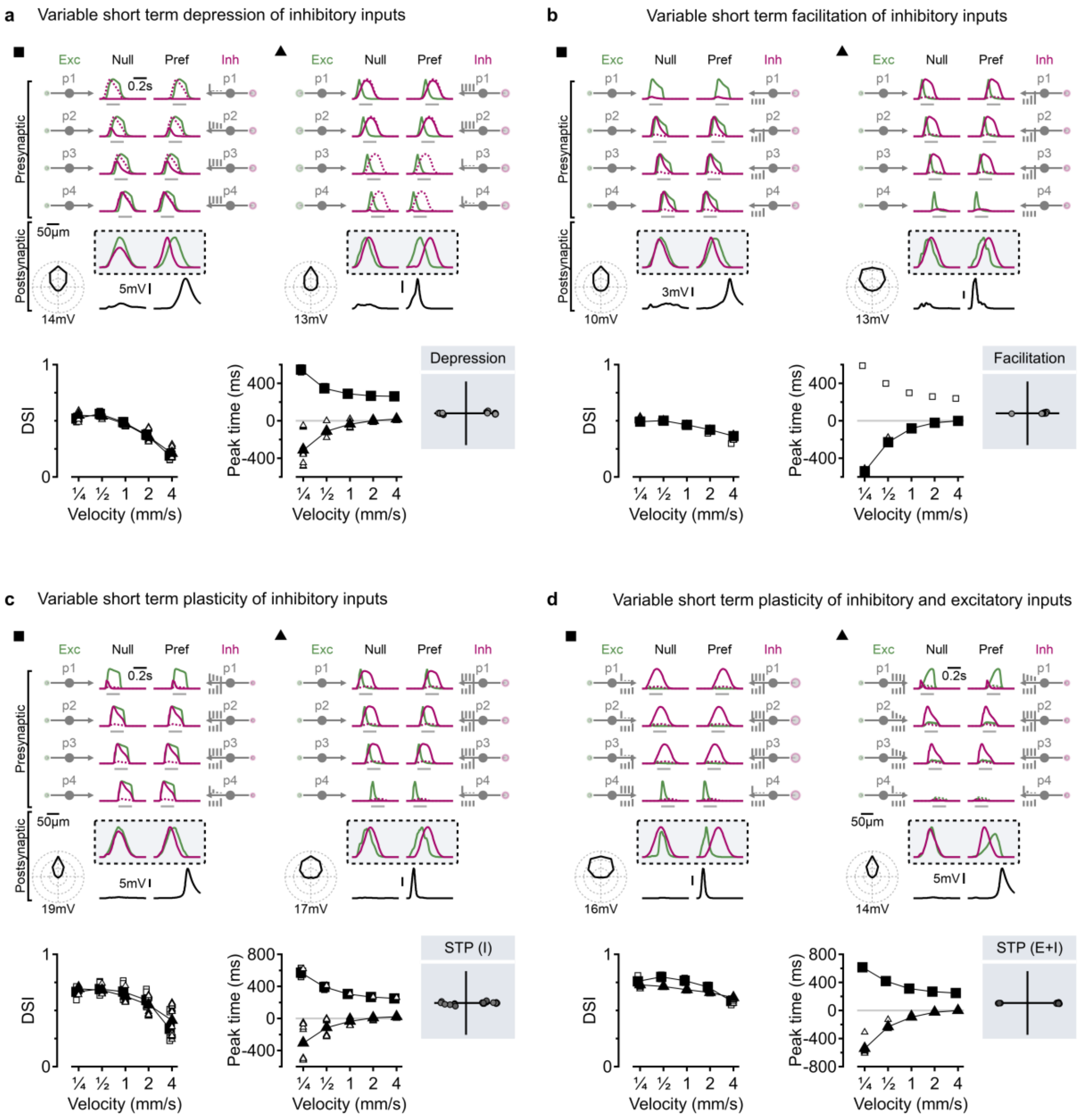


**Supplementary Figure 7: Models with short-term plasticity of the inhibitory drive.**

**a**, Top, two representative solutions to DS circuits with presynaptic inhibitory cells having different short-term synaptic depression levels. Dotted, original RF activation. Solid curves, signals after filtering with depressing synapses. Green, left – excitatory inputs. Magenta, right – inhibitory inputs. Bottom, velocity tuning (bottom left) and peak response times (bottom right). Model responses were clustered by their evolved parameters, resulting in a separation between B&L-like (triangle) and anti-B&L (square) interactions. Inset, illustration of the algorithmic solutions (computational primitives) seen in the models. See (**Fig. 6**) for details. **b-d**, As in (**a**), but for varying short-term facilitation (**b**), short-term plasticity of the inhibitory population (**c**), and short-term plasticity implemented in all synaptic inputs (**d**). N = 30 independently randomized simulation runs.

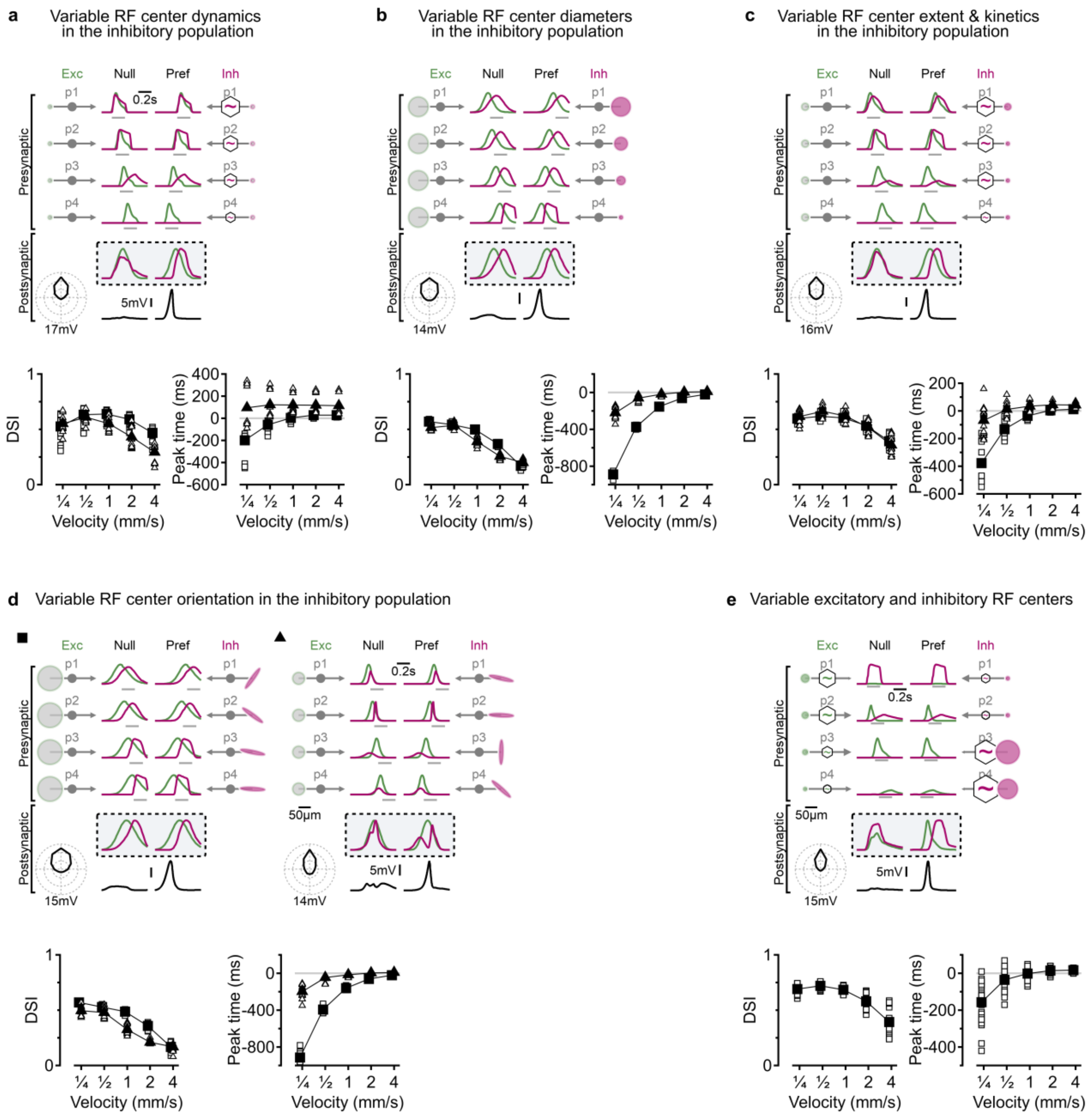


**Supplementary Figure 8: Models with varying spatiotemporal properties of inhibitory centers.**

**a**, Top, a representative solution to a direction selectivity circuit innervated by inhibitory cells with varying receptive field center dynamics. Green, left – excitatory inputs. Magenta, right – inhibitory inputs. Bottom, velocity tuning and peak response times for the preferred direction, separated into two clusters marked with squares and triangles based on the trained model parameters. **b-e**, As in (**a**), but for models with varying center-RF diameters of presynaptic inhibitory cells (**b**), variable RF size and kinetics (**c**), RF orientations (**d**), and variable RF size and kinetics of both excitatory and inhibitory drives (**e**). N = 30 independently randomized simulation runs.

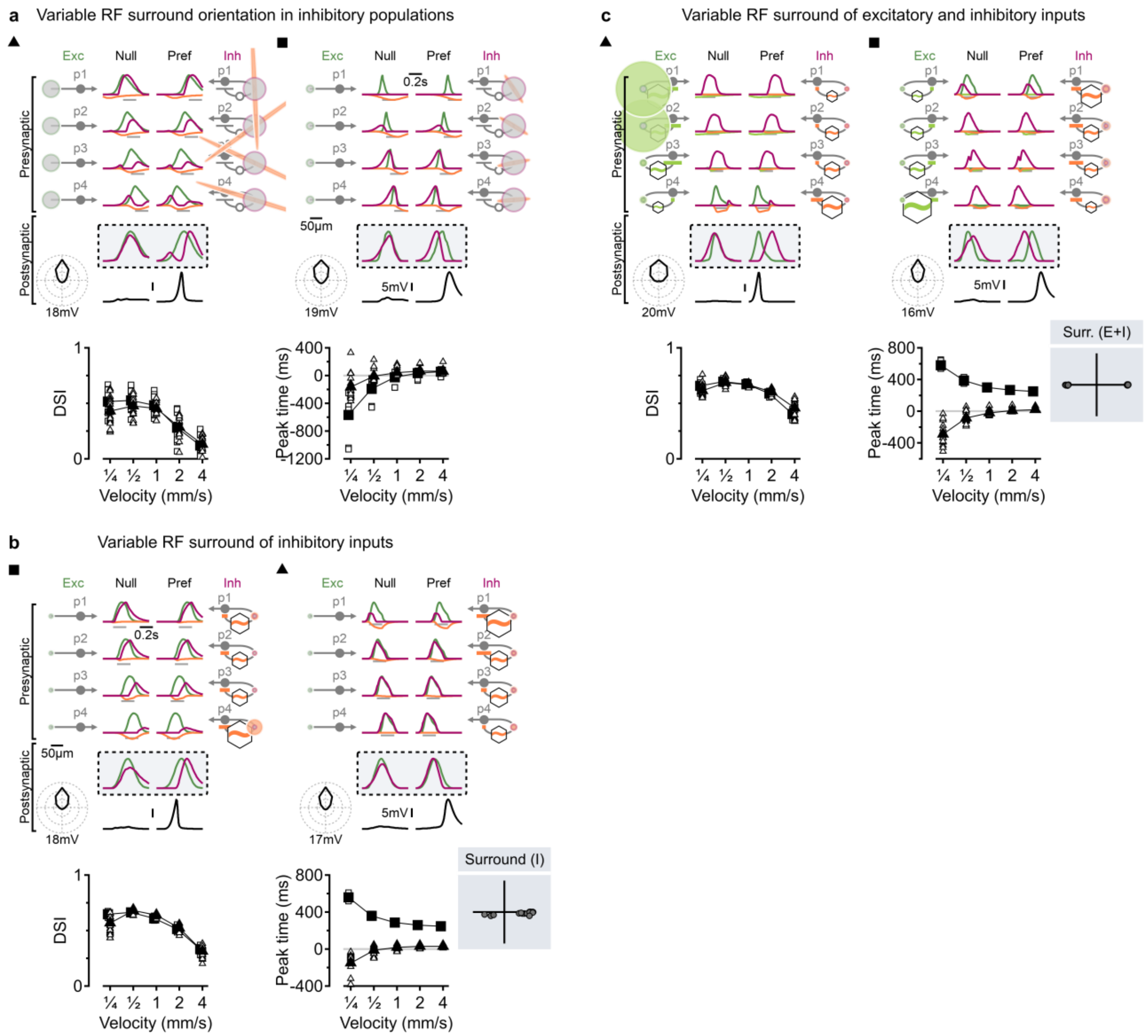


**Supplementary Figure 9: Solutions in models where the presynaptic inhibitory cells had variable spatiotemporal surround receptive field components.**

**a**, Top, representative solutions with B&L-like (triangle) and anti-B&L (square) interactions observed in a DS circuit innervated by inhibitory cells with varying RF surround strengths. Bottom, velocity tuning (left), and peak response times (right) of the two solution types. The range of algorithmic solutions is shown in (**Fig. 6b**). **b**, As in (**a**), but for a variable description of inhibitory cells' surrounds. Inset, computational primitives observed in this model family, axes, and color coding as in (**Fig. 6**). **c**, As in (**b**) for models free to vary in surround parameters of excitatory and inhibitory cells. Overall, flexible surround dynamics promoted robust DS via B&L and anti-B&L algorithms. N = 30 independently randomized simulation runs.

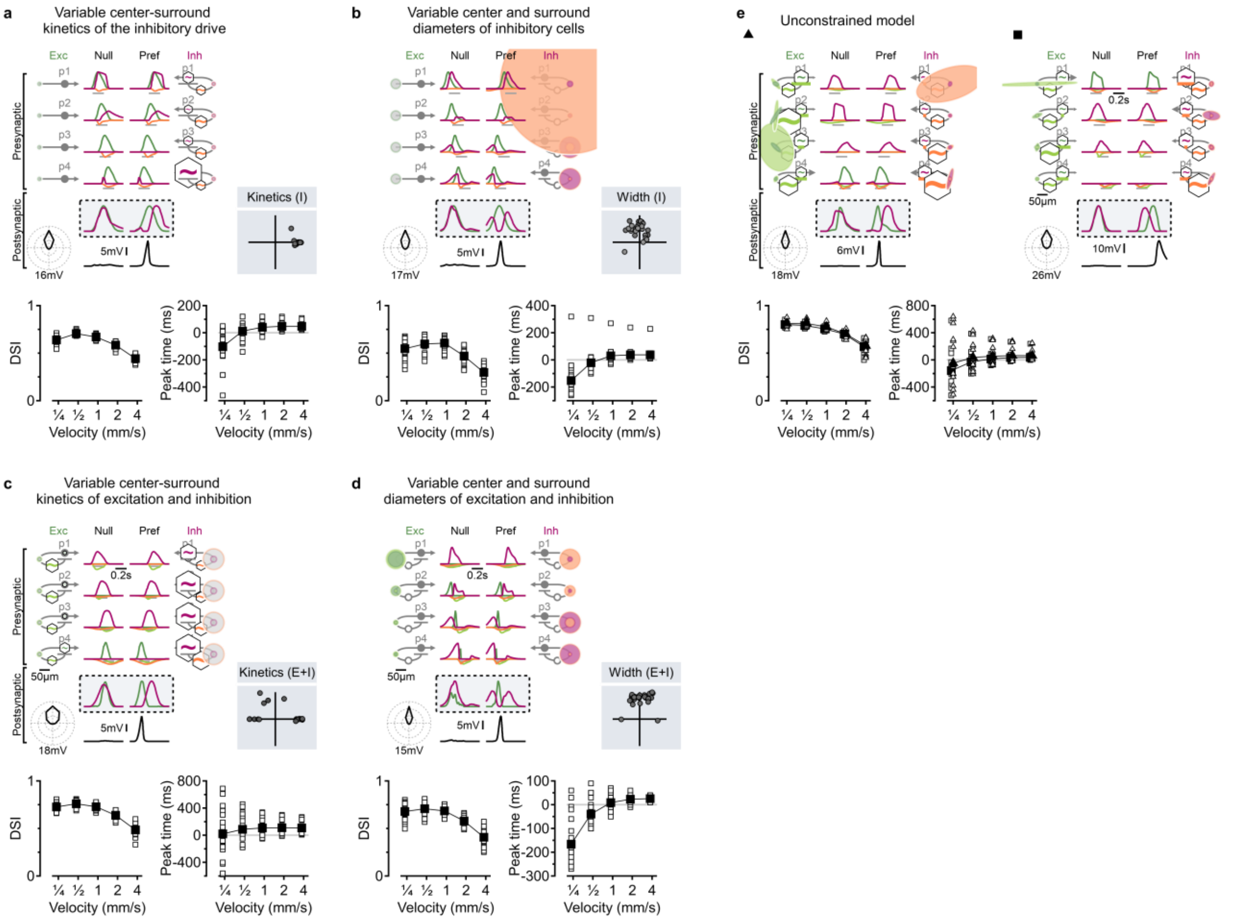


**Supplementary Figure 10: Solutions in models with multiple varying receptive field properties.**

**a-b**, Top, a solution with unconstrained center-surround kinetics (**a**) and sizes (**b**) of the inhibitory cells. Bottom, velocity tuning (left), and peak response times (right). Inset, the computational primitives observed in the models, axes, and color coding as in (**Fig. 6**). **c-d**, As in (**a-b**), but for varying excitation and inhibition. **e**, As in (**a**), but for models with unconstrained RF properties of excitatory and inhibitory populations exhibiting B&L (triangle) and anti-B&L (square) computational primitives. The extent of algorithmic solutions encountered in these models is depicted in (**Fig. 6c**). N = 30 independently randomized simulation runs.

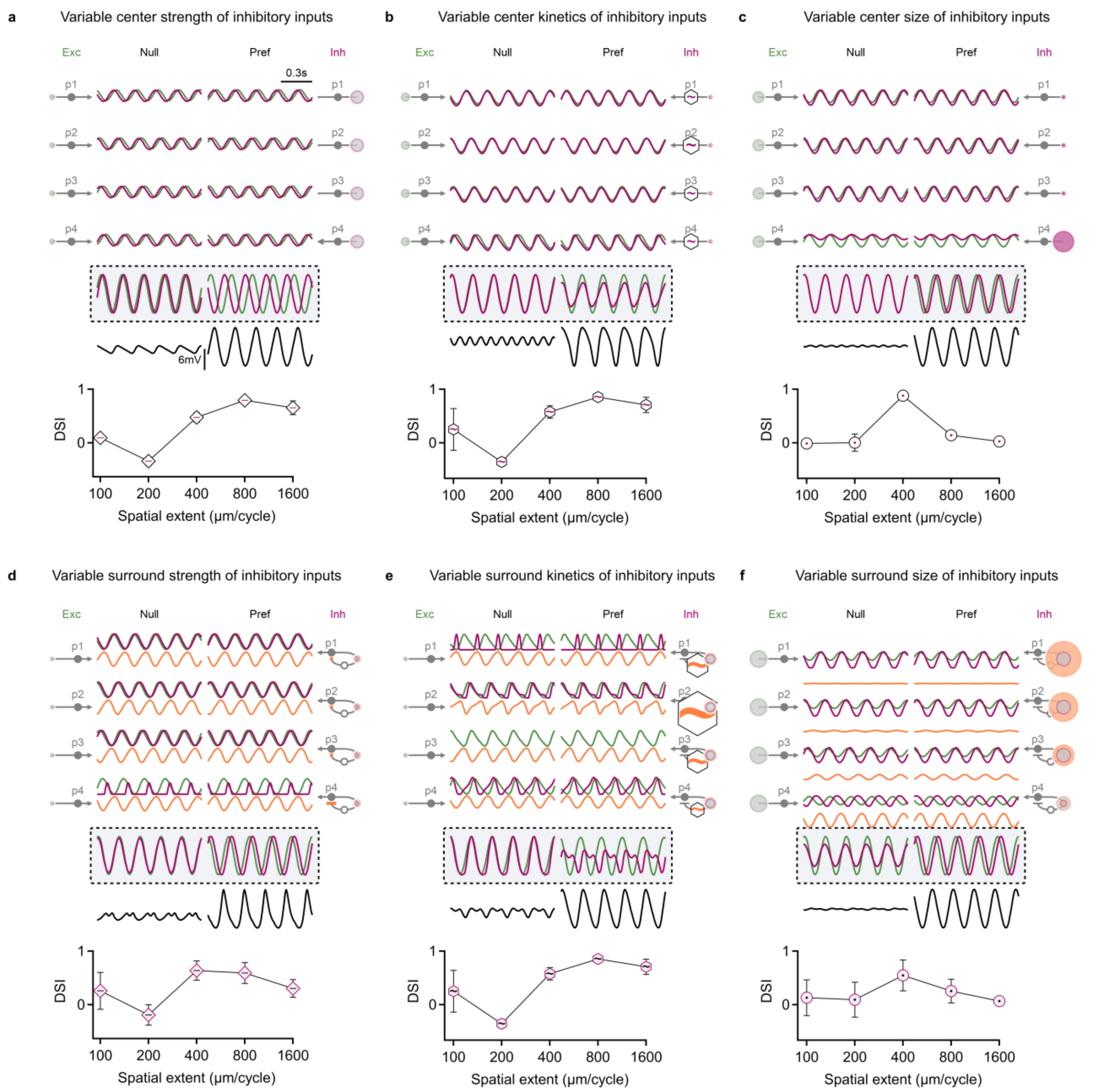


**Supplementary Figure 11: Detection of drifting grating motion direction with inhibitory inputs.**

**a**, Top, a representative solution to a circuit stimulated with drifting gratings (spatial extent = 400 µm/cycle; speed = 1 mm/sec; contrast = 100%) where presynaptic inhibitory cells were allowed to vary in their strength. Excitatory inputs had identical RF formulation (left, green). Magenta, right - inhibitory inputs. The inset shows cumulative excitatory and inhibitory conductance. Inhibition preceded excitation in the preferred direction, in accordance with anti-B&L logic. Black traces, membrane potential recorded at the soma. Bottom, spatial frequency tuning, error bars, SD, n = 50 indpendently seeded simulations. **b**, As in (**a**), but for variable RF center kinetics of inhibitory cells. In the example shown, the model evolved toward a tuned inhibition computational primitive. **c**, As in (**a**), but for variable RF center size and B&L implementation. **d-f**, As in (**a-c**), but for RF surrounds. Note the 'pause-in-inhibition' solution in (**e**).

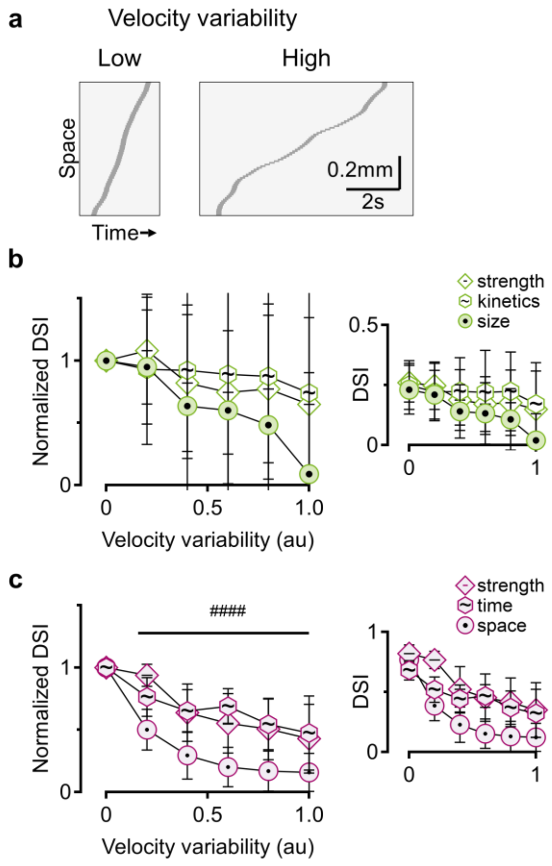


**Supplementary Figure 12. Differential resilience of surround direction selectivity solutions to stimulus variability.**

**a**, Space-time plots as in **Fig. 8a**. **b-c**, Summary of directional discrimination as a function of stimulus variability for excitation-only models (**b**) and in the presence of inhibition (**c**). DSI values were normalized to the no-noise baseline (inset, absolute DSI values). ####$p < 0.0001$; significant difference between noise-dependence slopes of variable inhibitory RF surround extents and other inhibitory configurations (ANOVA on linear regression fits, $n = 30$ independently randomized simulation runs). Error bars: standard deviation.

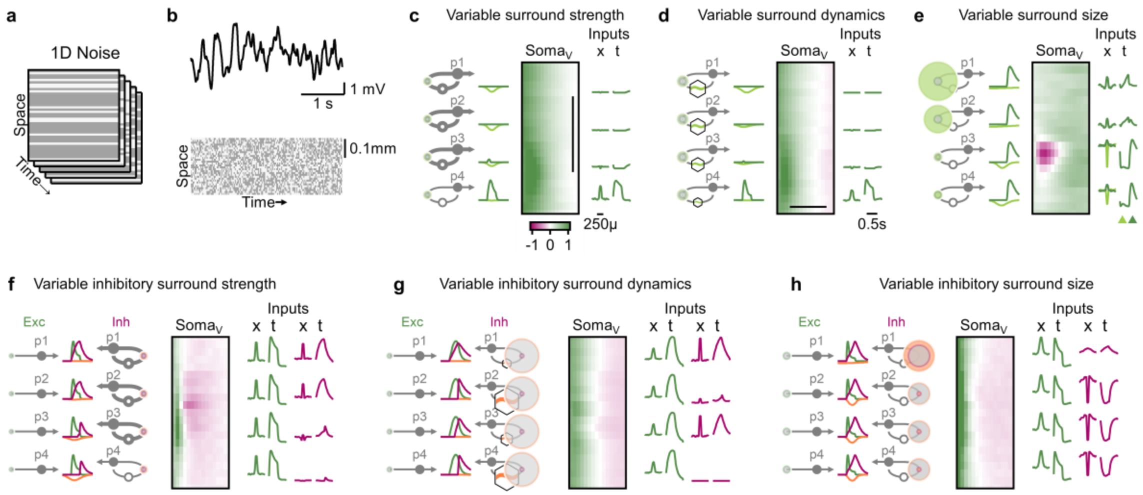


**Supplementary Figure 13. 1D noise stimulation can not reliably resolve the architecture of direction selectivity circuits with variable surround receptive field components.**

As in **Figure 9**, receptive field structures were assessed using oriented bars aligned with the preferred direction of motion. **a**, In each frame, individual bars were randomly assigned black or white contrast values to create a 1D bar noise stimulus. **b**, Representative membrane potential trace recorded from the DSGC soma (top) in response to 1D bar noise stimulation (bottom) in (**a**) model where motion selectivity arises from differences in surround strengths. **c**, Left: Ground-truth spatiotemporal profiles of four excitatory presynaptic populations from an evolved model with variable surround strengths. Temporal responses were derived from a full-field flash stimulus. Center: Space-time plot of the average postsynaptic response in DSGC following the appearance of a white bar at each spatial location. The vertical axis indicates spatial position; the horizontal axis shows time after stimulus onset. Color coding represents depolarization (green) and hyperpolarization (red). Scale bar: 250 µm, centered over the DSGC's RF. Right: Inferred RF properties of the presynaptic populations based on their responses to the 1D noise stimulus. Presynaptic analysis captures some circuit organization principles but does not reveal the role of surround. **d-e**, As in (**c**), but for models in which motion selectivity arises from differences in surround RF dynamics (**d**) size (**e**). Colored triangles in (**e**) indicate two different time points for which the spatial RF was analyzed. **f-h,** As in (**c**), for models in which DS is mediated by differences in inhibitory surround strength (**f**), dynamics (**g**), or size (**h**).

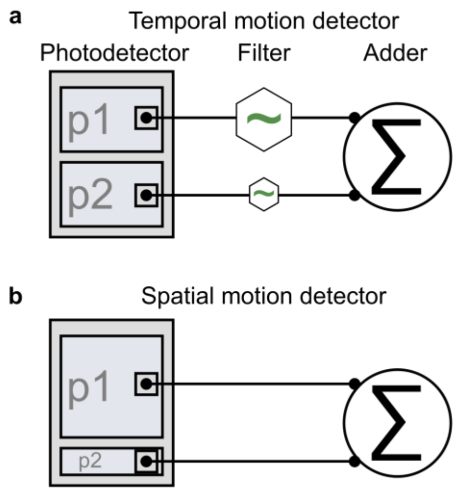


**Supplementary Figure 14. Possible implementations of artificial motion detectors.**

**a**, Schematic of a simple neuromorphic motion detector based on Hassenstein-Reichardt correlator logic. Two photodetectors (p1 and p2) feed into an analog integrator (adder). DS arises from differential temporal filtering: p2 is subjected to a delay or low-pass filter, producing stronger summation when motion engages the detectors in the upward direction. **b**, Similar schematic for a spatially based motion detector. Here, direction selectivity emerges from differences in photodetector size: p1 has a larger receptive surface area, while no temporal filter is applied. As demonstrated in this work, such spatial asymmetry can generate robust upward motion responses with selectivity comparable to the kinetic implementation.
